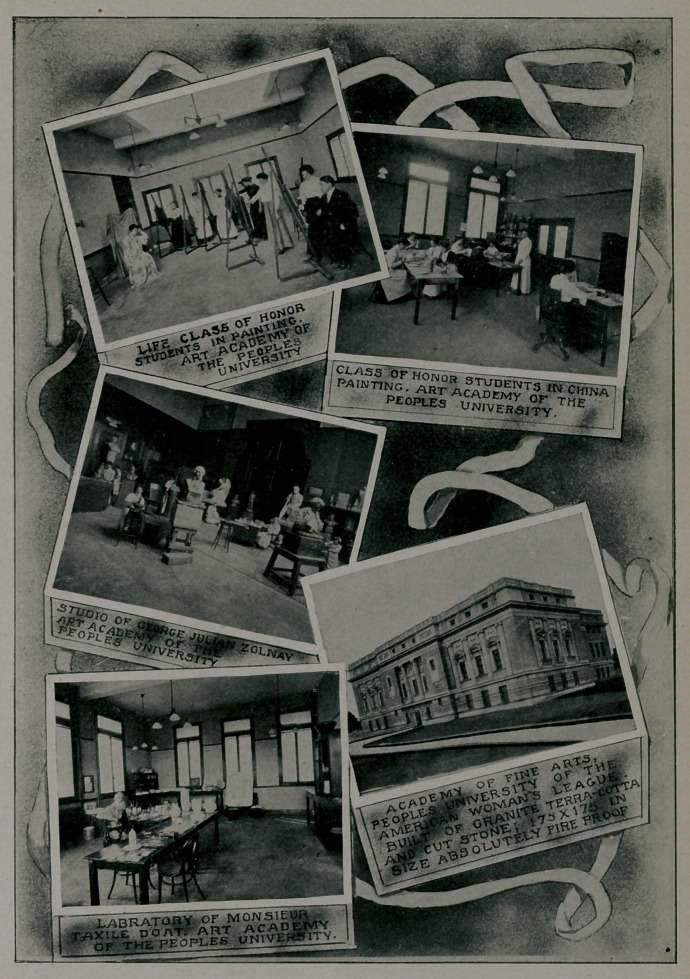# Book Reviews

**Published:** 1910-08

**Authors:** 


### BOOK REVIEWS.

DISEASES OF THE STOMACH AND INTESTINES. By Robert Coleman Kemp, M. D., Professor of Gastro-Intestinal Diseases, New York School of Clinical Medicine. Octavo of 766 pages, with 279 illustrations. Philadelphia and London. W. B. Saunders Company, 1910. Cloth, $6.00 net; half morocco $7.50 net.

In this we find a practical and workable book. Full enough it is to avoid ambiguity; brief enough to escape the pitfalls of pedantry.

Physiologically, it reflects fairly well the present-day viewpoint of the processes of digestion and assimilation, consequently its pathology and treatment are logical and orthodox.

Being personally acquainted with Professor Kemp, and having had ample opportunities to observe his methods, the reviewer is able to recommend this book as the intelligent and conscientious effort of an experienced gastroenterologist, whose clinical pictures are the fruit of experience; whose deductions are those of a scholar; whose therapeutic suggestions will yield satisfactory results when tried out in daily practice. G. M. N.

---

DISEASES OF THE EYE.—A Hand-book of Ophthalmic Practice for Students and Practitioners, by G. E. de Schweinitz, A. M., M. D., Professor of Ophthalmology in the University of Pennsylvania and Ophthalmic Surgeon to the University Hospital. With 351 illustration and 7 chromo-lithographic plates. Pp. 945. Price, cloth $5.00 net. W. B. Saunders Co., Philadelphia.

This classic work needs neither an introduction to the medical profession nor praise. The five previous editions have proved its unqualified success. The present volume has been thoroughly revised and affords a safe and complete source of information; it is well printed, well illustrated and well bound, as are all of Saunders' books.

---

ESSENTIALS OF PHYSIOLOGY, by Sidney P. Budgett, M. D., formerly Professor of Physiology in Washington University, St. Louis. Third edition, revised by Haven Emerson, M. D., Demonstrator of Physiology, Columbia University, St. Louis. Third edition, revised by Haven Emerdelphia and London. W. B. Saunders Company, 1909. Cloth, $1.00 net.

The third edition of this little book is arranged as the previous editions, with questions following each chapter which affords a useful aid to the student in thinking over what has been read.

---

DISEASES OF THE SKIN.—A manual for students and practitioners, by Alfred Slalek, M. D., Professor of Dermatology, University of Nebraska. Second edition, thoroughly revised; illustrated yith 47 engravings. Lea & Febiger, Philadelphia.

The book under review is one of the useful "epitomic series" of works published by this well-known firm. It presents the essentials of diseases of the skin in a manner somewhat more complete and in a more connected manner and yet not so voluminously as do large text-books. It has been revised up to date and provides a concise and useful manual on this subject.

---

NEPHROCOLOPTOSIS. A description of the nephrocolic ligament and its action in the causation of nephroptosis, with the technic of operation of nephrocolopexy, in which the nephrocolic ligament is utilized to immobilize both kidney and bowel, by H. W. Longyear, M. D., Professor of Gynecology and Abdominal Surgery, Detroit Post-Graduate Medical School. With 88 illustrations; pp. 251. C. V. Mosby Co., Publishers, St. Louis, Mo.

Longyear presents in this interesting monograph his views and studies regarding the importance of the nephrocolic ligament in nephroptosis. He believes he has discovered in this ligament the principal positive etiologic factor in dropping of the kidney and his reasoning is so clear and logical, backed by many incontestable facts, that he appears to prove in a convincing manner the truth of his theory. The unsatisfactory therapeutic and operative measures heretofore utilized in the management of this affection should lead both physician and surgeon to hail with delight a method of procedure at once reasonable as well as effective.

Longyear urges that we concentrate attention on the *beginning* of pathology and the entire involvement—not just the kidney. It seems quite reasonable to assume that an organ the size of the kidney would not of its own weight become displaced. Anatomical dissections have shown a ligament connecting the kidney with the colon. The colon drags down the kidney and in the operation of nephropexy success depends upon removing the cause i. e., fixing the nephrocolic ligament. This book is original from start to finish and is worthy of high praise.

### THE BARNES UNIVERSITY.

The Barnes University of St. Louis is now offering a course for medical students that should particularly appeal to those who desires to become practical physicians. The institution is now entering an ero of acknowledged prosperity. The elementary studies of the first two years, comprising what are known as the laborator ybranches, to-wit, anatomy, histology, embryology, physiology, physiological chemistry, inorganic and organic chemistry, bacteriology, pathological anatomy and pathological histology, surgery, pathology and clinical pathology, will be given by experienced teachers, each of whom will endeavor at all times to emphasize the direct and close relationship of laboratory science to clinical medicine. It is not the aim or desire of this institution to graduate research workers. That phase of medical education which tends to make practical, up-to-date and scientific physicians will be followed strictly throughout the course at the Barnes University.

The teachers in the elementary branches are all men who besides their laboratory training *know* clinical medicine and the difficulties and pitfalls in clinical diagnosis. Such teachers are unquestionably better qualified to teach *medical* students *medicine* than are the so-called non-medical laboratory teachers or even those who possess a medical degree and know nothing of clinical medicine. The aim of the Trustees of this institution has been to employ only teachers in laboratory subjects who can point out the importance of this or that matter as it arises in the elementary branches in relationship to the superstructure of medicine itself. The thought has ever been before the governing body of this institution to make *good* and *useful* doctors of medicine of every student from the time he enters school, not waiting until he has completed two years of so-called "ultra-scientic" laboratory work taught by men whose thoughts soar far above the practical demands of practicing physicians as is the case in many of the so-called "better schools" of medicine in this country.

The limitations of the four-year course of study make it obligatory on all schools that have in mind the training of practical physicians, the immediate need of teaching medicine to the young students once they have crossed the threshold; in other words to incorporate *medicine* into every step and procedure in the curriculum.

The Barnes University has also considerably strengthened its faculty by adding to it a number of excellent teachers of the clinical branches, medicine and surgery particularly. These teachers are thoroughly imbued with the need of developing *practical* men for *practical* work. Ample clinical facilities are at hand for such teaching. Besides the large out-clinic of the Barnes University. the institution also boasts of a hospital of some ninety beds' capacity in direct physicial connection with the medica lschool building where opportunity is given for bedside instruction to groups of students. This hospital also houses the material used for obstetrical teaching so that every student in the senior class has the magnificent opportunity of witnessing at least five obstretrical cases during his course. Clinical facilities of peculiar significance and importance for practically-taught students are also offered by Barnes University in the shape of opportunity to observe cases of infectious diseases especially the acute exanthematha, at the Ellen Osborn Hospital—the only private hospital in St. Louis which receives contagious diseases.

Barnes University recognizes all scientific advances that arise from time to time. It has therefore been deemed advisable that a full and comprehensive course be givn on immunity, the "vaccine" treatment of acne, septicemia, etc., the Wassermann and Noguchi reactions for syphillis. Steps are now being taken to organize a department for the administration of the Pasteur treatment for rabies at this institution.

All in all, Barnes University feels that it is well prepared to give students who enter its portals a full and complete course in medicine. The faculty feels that it is competent to teach and that the facilities are there for such teaching. The faculty is enthusiastic and determined to do its work in such a manner that it will have cause for naught else but pride in its end product—the full-bedged doctor of medicine graduated at the end of the designated time, four years.

---

### THE AMERICAN WOMAN'S LEAGUE.

### Its Plan and Purpose—Its Importance to Women of the South—Its Wonderful Opportunities For Educational Cultivation.

With its tens of thousands of members and over 700 regularly organized local branches or chapter, the American Woman's League may be considered one of the most useful of American institutions and one whose future offers prospects so bright that it takes second place to none. It is a simple business plan of co-operation between leading publishing concerns and local societies and clubs of women, and individual women. It makes no pretense of being anything but a business organization for the purpose of mutual benefit and profit. Its organization was first conceived and undertaken, in 1908, by the Lewis Publishing Company, of University City, St. Louis, one of the largest publishing concerns inthe world, which remains alone responsible for and in full control and direction of the plan, until its organization is completed, when the league will be permanently established under a trust agreement to be approved by the membrship itself.

Early in 1909, six other leading publishing houses were invited to co-operate under the plan, so far as the subscription end was concerned, and at the present time seventy-three others of the leading journals of the country have made application to join in the plan, which will ultimately be thrown open to all acceptable publications, on fair terms. . T.he. plan of The League consist of effecting a permanent national subscription or sales organization for reptuable publishers, one-half of the income going to the publishers who receive fifty per cent. of their subscription price, net cash; and the other half to this organization known as the American Woman's League.

It is estimated that the annual gross subscription income of the leading magazines and journals of general circulation in this country exceeds $60,000,000. It is also estimtaed that it requires the expenditure by the publishers each year of the greater part of the $60,000,000 to secure and renew this subscription by means of endless schemes, premiums, commissions, prizes, contests and advertising matter. A comparatively few years ago, all journalism was entirely on a subscription basis, advertiesments not being accepted at all by reputable publishers. Today it is on an entirely advertising basis, the subscription income playing only a nominal part. With tens of millions of dollars invested in their manufacturing plants, producng a better article for a lower price than any other industry, the publisher, as a manufacturer, has no permanent national wholesale and retail sales organization such as other industries have in the jobber and retailer, but goes direct to the consumer scattered from end to end of the land, at a selling cost, in many cases, greater than the selling price.

The center of the American Woman's League movement—its capital city, so to speak—is University City, St. Louis, a separate municipality of some 2,000 acres, incorporated and laid out some years ago, before it had become the finest residence section of St. Louis, owing to the growth of that city. The officers of the League are also the municipal officers, the president being the mayor. Here are located the magnificent institutions of the Lewis Publishing Company, the largest and finest publishing institutions in the world, and here are rapidly being erected the other great institutions of the League. It is designed to make University City the most beautiful and model municipality in America, and much has already been accomplished along these lines.

The principal benefits of membership are:

First, the free right to all courses of instruction in its great schools, university, and art institute.

Second, the use of its postal library and phonographic library, a phonographic instrument of superior make being supplied members in their homes as well as chapters.

Third, a National Woman's Exchange, of which each local chapter is a branch, for the marketing of woman's handiwork and the products of the League. . .

Fourth, a loan and relief fund for loans at low interest for home building. They bear no interest when used to relieve distress.

Eventually, with the accumualtion of a reserve orsurplus, it is proposed to erect and equip the finest home or retreat and orphanage in America, where a member, destitute and alone in old age, may be cared for; also for the care and education of orphaned children of members.

## Figures and Tables

**Figure f1:**